# A Composite Method Based on Formal Grammar and DNA Structural Features in Detecting Human Polymerase II Promoter Region

**DOI:** 10.1371/journal.pone.0054843

**Published:** 2013-02-20

**Authors:** Sutapa Datta, Subhasis Mukhopadhyay

**Affiliations:** Department of Biophysics, Molecular Biology and Bioinformatics and Distributed Information Centre for Bioinformatics, University of Calcutta, Kolkata, West Bengal, India; Virginia Tech, United States of America

## Abstract

An important step in understanding gene regulation is to identify the promoter regions where the transcription factor binding takes place. Predicting a promoter region *de novo* has been a theoretical goal for many researchers for a long time. There exists a number of *in silico* methods to predict the promoter region *de novo* but most of these methods are still suffering from various shortcomings, a major one being the selection of appropriate features of promoter region distinguishing them from non-promoters. In this communication, we have proposed a new composite method that predicts promoter sequences based on the interrelationship between structural profiles of DNA and primary sequence elements of the promoter regions. We have shown that a Context Free Grammar (CFG) can formalize the relationships between different primary sequence features and by utilizing the CFG, we demonstrate that an efficient parser can be constructed for extracting these relationships from DNA sequences to distinguish the true promoter sequences from non-promoter sequences. Along with CFG, we have extracted the structural features of the promoter region to improve upon the efficiency of our prediction system. Extensive experiments performed on different datasets reveals that our method is effective in predicting promoter sequences on a genome-wide scale and performs satisfactorily as compared to other promoter prediction techniques.

## Introduction

Promoters are the key regulatory elements known to regulate the initiation of transcription in both eukaryotes and prokaryotes. In a DNA sequence, promoter is the region that appears immediately upstream of a Transcription Start Site (TSS) that performs a crucial role in initiation of transcription and is responsible for binding of RNA polymerase [1], [2]. Therefore, an in-depth characterization of this region can provide a deep insight about the transcription initiation and gene function. Also, the prediction of this region can be very successfully used to discover the genes that are often missed out by several gene prediction tools. Moreover, proliferation of eukaryotic sequences due to second generation sequencing project requires a fast as well as an accurate method that can predict and characterize the regulatory regions in a genome. Significant progress has been made towards developing methods for recognizing the promoter regions of DNA sequences, yet it still remains an interesting and a challenging problem from the view point of bioinformatics.

The promoter region is divided into three parts: (1) the core promoter, (2) the proximal promoter and (3) the distant promoter. As an illustration of the proposed method, we are focusing only on predicting the core promoter region which extends typically 50 bp (base pairs) upstream to 50 bp downstream of the TSS [2]. It is known that the promoter sequences possess some special signatures that can be exploited to distinguish them from the rest of the genome sequences and these entire set of promoter properties are known to be conserved within a species. Needless to say, there exists a significant exception, which makes promoter recognition a complex problem. Nevertheless, a few core promoter elements have been detected, of which the most common elements are CpG island, TATA box, initiator (Inr), downstream promoter element (DPE) and TFIIB recognition element (BRE) [2] but these promoter elements may not be universal. A statistical survey on ∼10000 predicted human promoter data reveals that among these four promoter elements Inr is the most common and usually occurs about half of the human promoters [3]. DPE and BRE found nearly one fourth of the promoters and TATAbox found roughly one eighth of the human promoter. A quarter of all promoters do not contain none of the promoter elements. About ∼60% of the human core promoter falls near a CpG island a short stretch of DNA having a high G+C content and a high frequency of GC dinucleotide compared to the bulk DNA. Certain combinatorial nature exist among CpG island and certain promoter elements. Such as TATAbox are more common in promoters lacking the CpG island nearby whereas BREs are more common in the promoters that are associated with CpG island. Utilizing these known signatures, a number of computational algorithms have been proposed. Among them the methods, prove to be very useful in predicting promoters in vertebrates are based on CpG island near TSS [4]–[6]. A host of computational methods have been proposed based on the recognition of TATA box [7], [8], CAAT box [9], the recognition of the presence of specific transcription factor binding sites (TFBs) [10]–[12], utilizing a pentamer matrix [7], and locating oligonucleotides [13]. But these methods typically utilize heterogeneous local sequence compositional signals represented by the promoter elements alone; thus they cannot identify the true promoters and reports in a high number of false positive instances. Other promoter prediction methods use statistical properties of the core and proximal promoters [14], [15], similarities between orthologous promoters [10] and information from mRNA transcripts [16]. Relatively new developments are based on machine learning techniques, such as discriminant analysis, Hidden Markov Model, Artificial Neural Network, Support Vector Machine and Stochastic Context Free Grammar [17]–[21]. Exploiting the structural properties of DNA sequences to predict the promoter regions have also been employed. A number of studies have revealed that the eukaryotic core promoters have distinct structural properties that distinguish them from other non-promoter regions. Thus it becomes feasible to predict promoter region from structural perspectives [22]–[26].

Motivated by these methods, we present a new method for predicting *de novo* the promoter regions. Our method is based upon formal grammar and structural properties of promoter sequences.

A formal language is a language that is defined by precise mathematical or machine processable formulas. A formal grammar or syntax is that generates the language. The syntax i.e. the grammar of a formal language can be formally presented by specifying a set of nonterminal symbols or variables, a set of terminal symbols and a set of production rules of the form S→a or S→AaB, where S, A, B are the nonterminal symbols and a is the terminal symbol. The recursive application of production rules beginning from start symbol S can generate a set of string containing only terminal symbols, which is the language generated by the grammar. Here we have developed a context free grammar that has been used to design and verify the structure of a promoter sequence. The term “context free” implies that there should not be any terminal symbol on the left hand side of the production rules.

A Context Free Grammar (CFG) G1 is a 4 tuple, defined as
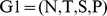
where N is a set of nonterminal symbol, T is the set of terminal symbol, S is the start symbol and P is a finite set of production rules where S belongs to the nonterminal set.

Many researchers have shown that formal language theory is an appropriate tool in analyzing various biological sequences. A recent article gives a brief description of most of these works, attempted to analyze the biological sequences using formal language theory [27].

Computational grammars have been used in modelling and predicting transcription binding site [28], associating genes with their regulatory sequences [29], predicting RNA folding [30], secondary structure of RNA molecule [31]–[33], genes and biological sequences [34], [35], syntactic model to design genetic constructs [36]. Grammatical models have also been developed with the goal of designing new antimicrobial peptides [37]. Nowadays discovery of grammar rule from biological data set i.e. grammar inference is an active field of research [38] and has been used in predicting larger than gene structure [39]. In recent years Stochastic context free grammar has been used in predicting protein sequences [40].

Our method uses a set of CFG rules and structural features of the sequences to predict true promoter and non-promoter sequences from a set of mixed database and also to identify the promoter regions on genome-wide scale. Instead of using any grammar inference algorithm to obtain the grammar rules from any training set we have utilized pre-existing biological knowledge to generate the grammar rules. In this case, the grammar rules are representing only the promoter sequences. So the sequences generated using these grammar rules are predicted as the putative promoters. Most the existing promoter prediction methods doesn't give any idea about the presence or absence of the functionally important elements of the promoter regions and also have not showed any relationships between these elements. Also these methods have not showed how the combination of primary sequence features and structural profiles of the promoter region enhances the prediction accuracy. Here, we are using the grammar rules to obtain an inside view of the promoter regions i.e., to recognize the specific functionally important fragments or elements that a promoter region can posses and also then utilizing the structural features to verify and improve upon the promoter prediction performance.

## Materials and Methods

### 1. Materials

We have extracted 14000 promoter sequences from DBTSS database (http://dbtss.hgc.jp/), each of which is 1201 bp long ranging from −1000 to +200 positions relative to the +1 position of transcription start site. We have used release hg19 of human genome for the analysis. For this purpose, we used the RefSeq genes downloaded from the UCSC table browser (http://genome.ucsc.edu/cgi-bin/hgTables?command=start) [41]. This set includes 25000 unique gene sequences. We have also generated a human non-promoter sequence set containing 25000 random fragments of 1201 bp long by selecting intron sequences, exon sequences and 3′ UTR regions sequences to avoid any sequence bias of coding DNA. To compare the performance of our method with other currently available promoter prediction programs, a comparative evaluation is done on another dataset- the CAGE dataset which has a wider coverage on genome sequence. CAGE dataset is obtained through the cap analysis gene expression (CAGE) technique and is retrieved from Riken Institute website using FANTOM3 project (http://fantom.gsc.riken.go.jp/) [42]. As described earlier in the previous studies [22], [24]–[25], tag clusters with at least two mapped tags on the same genome location are considered for analysis in our studies. These tags are then mapped to human genome sequence to obtain 180000 unique human TSSs. The whole human genome (hg19) is retrieved from UCSC Genome Bioinformatics site (http://genome.ucsc.edu/) [43].

### 2. Methods

#### 2.1 CpG islands and CpG counts

In most of the vertebrates promoter regions are localized by an atypical structure, the CpG island (CGIs). CGIs are unmethylated structures of DNA, spanning about 200 bp or more and are characterised by a high G+C content of more than 50% and the observed/expected CPG ratio of 0.60, relative to the bulk DNA. CpG islands are found in approximately half of the whole mammalian promoters [44]–[46] and also estimated to be associated with more than about 60% of human promoter [47]. For this reason, Pederson *et* *al* [48] suggested that CpG island could represent a good global signal for locating promoters across genomes. At least for mammalian genomes, CpG islands are good indicators of the presence of a gene. In our prediction system, we have used the presence or absence of CpG island as a distinguishing feature for a DNA sequence.

The G+C content (GC-Con) and observed/expected (o/e) ratio are defined as follows:
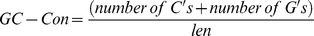



and
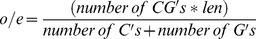
where len represents the length of one segment of a DNA sequence.

#### 2.2 Features of the DNA sequence

We have focused our attention only on core promoter regions of human DNA sequences that extend 50 bp upstream to 50 bp downstream of the TSS. The few core promoter elements have been detected as of now among which the most common elements are TATABOX, Initiator (Inr), Downstream Promoter Element (DPE) and TFIIB recognition element(BRE) [2]. We have used a CFG to support the design of core promoter region containing these promoter elements and feature represented by CpG island. Instead of inferring the production rules using any machine learning techniques from a set of training data, our production rules are based on pre-existing biological knowledge such as, if a sequence contains tatabox that occurs between −10 to −50 bases upstream relative to TSS, then it is considered to be a true TATABOX.

#### 2.3 Nonterminal set

In constructing the grammar for core promoter recognition, we begin by identifying the syntactic elements used to organize the signature elements in a promoter region which represents the CFG variables or nonterminal symbols along with a start symbol and other nonterminal symbols. The syntactic elements within a promoter region are listed in **Table** **1**. We use only 6 syntactic elements, each of them are represented by a single capital letter. The orientation of constructs is from the left to the right. Nonterminal set consist of one start symbol S, the variable from which all the derivation initiates. Two other variables S1 and S2 are also included to the nonterminal set to generate the promoter sequences having different sequence signatures. The nonterminal set includes variable T corresponding to Tatabox, variable I corresponding to Initiator element and variables D, B, G, C, representing DPE element, BRE, Gap and CpG island respectively.

**Table 1:** Syntactic elements or the nonterminal elements for human promoter region.

T→ TATA-box
I→ Initiator
D→ DPE
B→ BRE
C→ CpG island

G→ Gap i.e., parts of DNA sequences that are not of our interest or their significance is still not known. Here a gap in the grammar rule defines some length of bases flanked by two subsequence of interest. In the grammar rules, the symbol G (any) is used to specify a gap of indefinite length whereas when gap length is known to be within a range, gap (#Lower Limit, Upper Limit#) is preferred.

For the specific CFG presented in this manuscript, the nonterminal set is given by N = {S, S1, S2, T, I, D, B, G, C}.

#### 2.4 Terminal set

The next step in developing the grammar is to recognize the terminal symbols. The terminal set is composed of regular expressions describing the syntactic elements present in the nonterminal set. Here we have considered regular expressions as a symbol to represent the terminal symbol set. A credit assignment algorithm [49] is used to infer these regular expressions from a set of sample sequences. To do so a set of regular expressions is taken for each of the syntactic elements except the CpG island (presence of CpG island is obtained by the method described above) and Gap (Gap is identified as any sequence between two syntactic elements). Strengths associated with each of these regular expressions are calculated. The system analyses the test sentences comprising of positive and negative instances (S^+^ and S^−^) and for each of these regular expression, a credit assignment module assigns a score. As positive instances, we have taken the true promoter sequences from region −100 to +50 relative to +1 position from Eukaryotic Promoter Database(EPD) and the negative dataset comprises of non-promoter sequences of length 151 bp taken from EMBL and EID database. Regular expressions with highest score are assigned as the putative terminal symbol for that particular syntactic element or nonterminal symbol.

The algorithm consists of the following steps:

1. A set of regular expressions is randomly generated for one syntactic element.
2. All the sequences in S^+^ and S^−^ are searched for those regular expressions.
3. Terminate if all the sentences are correctly searched or all the regular expressions are being used.
4. Compute the strength of each regular expression.
5. Take the regular expression with highest strength.
6. Repeat the process for the other syntactic elements.

The strength of i^th^ regular expression is calculated by the expression:
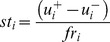
where *u_i_^+^* is the number of times the i^th^ regular expression is used for correct recognition (i.e., accepting sentences in S^+^ and rejecting sentences in S^−^) and u_i_^−^ is the numbers of times the i^th^ regular expression is used for incorrect recognition (rejecting sequences in S^+^ and accepting sequences in S^−^) and *fr_i_* is the number of times the i^th^ regular expression is used.

The final regular expression that represents the terminal set of the syntactic elements is shown in **Table** **2**.

**Table 2:** Regular expressions of different promoter elements which give the terminal set.

Promoter Elements	Regular Expressions
1. TATA-box	ta[ta][ta][tag][ta]
2. Initiator	[ctg][ctg]a[atgc][at][ct][ct]
3. BRE	[gc][gc][ga]cgcc
4. DPE	[ag]g[at][ct][cag]
5. Gap	[atcg]+

The possible sequences generated from each of the regular expression representing each part or syntactic elements are included in a parts library where each of the sequences representing each part is indexed by a unique identifier. For example, the regular expression of TATAbox is ta[ta][ta][tag][ta], from which 24 matching sequences can be generated that represents the terminal symbols of TATAbox. Terminals t1 to t24 represents tatabox in the part library whereas b1 to b8 would point to the BRE elements etc. The terminal symbol representing the presence of CpG island is represented as c0 and Gap as g0.

#### 2.5 Production rules

In the last step of developing the grammar we have designed some grammar rules. As stated earlier, we have used pre-existing biological knowledge to design these production rules. **Table** **3** shows a list of production rules or grammar rules for generating a putative promoter sequence. In designing the human promoter region the grammar rules are utilized in successive steps. The process begins with the start symbol “S”. Several rules can be applied to rule R1 to generate a promoter region of a sequence such as, rule R2 can be applied to R1 to generate those promoter sequences having CpG island and no TATAbox and Initiator. Alternatively, rule R3 and rule R6 can be applied successively to R1 to generate sequences having CpG island, TATAbox and Initiator or rule R5 can be used to generate promoter sequences having CpG island, BRE or TFIIB recognition element, TATAbox and Initiator and so on. Similarly, rule R4 can be used for generating sequences without CpG island. The last step of the design process involves the transformation of the nonterminal variables to the terminal symbols. Production rules R14 to R19 of the form variable→ terminal1|terminal2|... are indicating that all the nonterminal variables can be transformed to any of the terminal symbols separated by |. For instance a variable I (initiator) can be transformed into any of the terminal sequences representing initiator in the part library. The design process completes when all the nonterminal variables are transformed into the variables. A sequence is represented by a series of terminal symbols. We can predict that the sequences generated by the grammar are the putative promoter sequence. A survey of existing literature reveals that a very small amount of mamalian promoter contains these primary sequence features [9] such as TATAbox, BRE, Initiator and DPE. Another complexity exists as the length of the transcription factor binding sites in promoter sequences are not fully conserved and differs quite drastically. There exists a probability of finding these regulatory features elsewhere in genomes outside the promoter region, resulting in a generation of high degree of false positive instances. About more than half of the mammalian promoters contain CpG island making it an essential feature in predicting a promoter region. So, we have considered CpG island as an important feature while constructing the proposed grammar rules. But recognizing the promoters that do not contain CpG island becomes a non-trivial task. To eliminate the generation of spurious positive instances, we consider the structural features of promoter region for correctly predicting the true promoter sequences.

**Table 3:** Production rules of grammar G1 generation the promoter region of a human DNA sequence.

R1. S→ G S1 G
R2. S1→C
R3. S1→ C S2
R4. S1→S2
R5. S2→ B G(10,30) T G(10,40) I
R6. S2→ T G (10,40) I
R7. S2→ B G (10,30) T
R8. S2→ T G(10,40) I G(15,37) D
R9. S2→ I G(15,37) D
R10. S2→B G(10,30) T G(10,40) I G (15,37) D
R11. S2→ I
R12. S2→B G (10,50) I
R13. S2→T G (10,40) I G (15,37) D
R14. T→t1|t2|...
R15. I→I1|I2|...
R16. D→d1|d2|...
R17. B→b1|b2|...
R18. G→g0
R19. C→c0

#### 2.6 Structural Profiles

To calculate the structural profiles of a DNA sequence, the nucleotide sequences are converted into sequences of numerical values by replacing each di or tri nucleotide (depending upon the physio-chemical features used) with its corresponding structural feature values. The conversion table, representing different structural features are obtained from experiments and are summarised by Florquin *et* *al* [23]. For smoothing the raw profile, we use a sliding window approach with a step size of 1 and a window size of 3 nt respectively. After the window slides through a sequence, a vector of structural values is generated as the output. In this communication, propeller twist, bendability, nucleosomal positioning preferences, DNA denaturation, Zdna, Basestaking energy, bDNAtwist and Aphilicity scores of all observed di or tri nucleotides in sliding windows are calculated. Since all of these properties are calculated from conversion tables using di or tri nucleotide, it is assumed that these properties are exactly the same as the nucleotide sequence and do not give any extra information. However, several studies has shown the correlation between different properties and their main conclusion is that the properties are largely independent [50], [51]. According to Abeel *et* *al*, human core promoter region has a distinct structure that stretches over quite a long distance and it shows either a large peak or a cleft near TSS region [22]. It is thus reasonable to assume that in a promoter prediction technique, the region with a large peak or a cleft is most likely to contain a TSS.

## Results and Discussion

**Figure 1:**
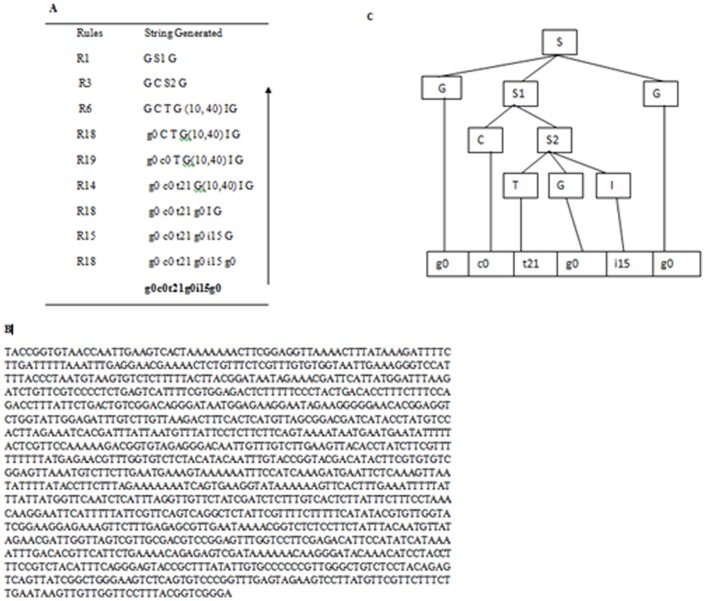
Process of generating the start symbol from input sequence using successive application of the grammar rules. (A) The strings generated by successive application of production rules to guide the design of a promoter region (B) The sequence of a promoter region composing of the parts of the syntactic element for verification (C) the LR parse tree for generating the symbolic description of the sequence using grammar rules.

To construct the sequence, we apply a set of production rules starting from S that generates a sequence with a structure consistent with the grammar rule. It is very necessary to construct the DNA sequence starting from S through the application of grammar rules to know whether or not a specific sequence can be generated by a given grammar. Here parsing is described as the process of going from a terminal DNA putative promoter sequence upto reach the final S symbol. By parsing a sequence, it is possible to verify the design of an input sequence those are generated by a systematic process using grammar rules. Here, we have used promoter and non-promoter sequences as input and if the input sequences are generated by the above mentioned grammar rules, then we can predict it as a putative promoter sequence. To do the parsing of the input sequence, first we have undertaken a lexical analysis of the given sequence to transform it in a number of parts or tokens, where each token represents a signature in the sequence such as Tatabox, Initiator, DPE, BRE and Gap sequences. We take the sequences from the part library except the regular expression for gap or G. Sequences from the part library are compared with start or leftmost sequence of the input. If the part does not match the start of the sequence, the next part in the list is evaluated and so on. At the end of the process, if no part matches the beginning of the input sequence, then the beginning sequence is considered as the region that represents the gap sequence and the rest of the sequence is analyzed in the same way. At the end of the scan, it may so happen that no part matches the input sequence. In that case the input sequence is rejected which means the input sequence cannot be tokenized and the sequence is considered as a non-promoter sequence. It may also be possible that several matches of a part will be found in a sequence. In that case, all the matches are recorded as the multiple interpretation of the token representing that part in the part library. Some of these tokens are true promoter signatures but some others are coincidental matches. We shall consider those tokens, starting from which we can derive the start symbol (S) according to the grammar rules. For the CpG island, we have only considered the presence or absence of CpG island in the input sequence and the presence of CpG island is denoted as c0 as the token of CpG island. Next, we have developed a customised parser that parses the input strings in a left to right bottom up manner by converting the input string into a series of nonterminal variables to obtain the start symbol S through the production rules in **Table** **3**. Finally, the resulting sequence is processed according to the order of the production rules. If we can annotate the start symbol S starting from the input string through the production rules, then the sequence is accepted as a promoter sequence. **Fig.** **1** illustrates the process of generating the start symbol from input sequence using grammar rules.

Precision, Recall and F-measure are used to evaluate the system's performance towards its predictive efficacy. The true promoters are considered as positive data and true non-promoters are considered as negative data. For the data, which are predicted as positive, the real positive ones are called true positives (TP), while the others are called false positives (FP). For the dataset which are predicted as negative, the real positive ones are called false negative (FN), while the others are called true negatives (TN). The formula of precision, recall and F-measure are given below. 
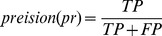
, 
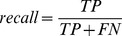
 and 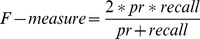
_._

In order to validate our method, we have used the grammar rules in **Table** **3** to annotate the test sequences. Test sequences comprise of 25000 true promoter sequences taken from UCSC table browser for RefSeq gene sequences of hg19 human genome and same number of non-promoter sequences taken from EID and EMBL database. Non-promoter sequences are combination of sequences from Intron, Exon and 3′ UTR regions of DNA sequences. Each of these sequences is 1201 bp long. For true promoter sequences taken from UCSC table browser, we have considered the region between 1000 bp upstream and 200 bp downstream of 5′ UTR as true promoter region and retrieved it. Of these sequences, the grammar rule can annotate 66.7% true promoters. Among true promoters, 54.7% sequences obey the rules that contain CpG island and remaining 12% data don't have CpG island. Remaining 33.3% true promoter sequences cannot be generated by the grammar rules. So there exist a high percentage of false negative instances. Further, we have taken the non-promoter sequence dataset among which 71.23% true non-promoters are truly predicted and the remaining 28.77% data are predicted as true promoters showing a high percentage of false positive instances. To validate the result, we have also taken the sequences from dbtss database. Here, we have considered the same number of non-promoter sequences as in dbtss dataset. Among true promoter sequences taken from dbtss database, 57.25% sequences are generated by grammar rules and 42.75% data cannot be generated using grammar rules showing the existence of a high percentage of false negative instances. To remove the false positive and false negative instances, we have incorporated the structural features of the promoter regions in a DNA sequence. According to several previous studies, human core promoter adopts a specific structure that stretches over a long range along the sequence i.e., it adopts to either a large peak which represents highly stable region or a cleft representing highly unstable region near the TSS site in the DNA sequence [22]. Abeel *et* *al* has shown in their study [22] that over a long range distance in the DNA sequence, the structural profile either gradually decreases or increases a few hundred base pairs in the upstream and downstream region of the TSS site with a high peak or cleft at TSS. Among the structural profiles Bendability, Nucleosomal position, Bending Stiffness, Propellertwist, DNADenaturation and ProteinDeformation shows a high peak near TSS region and Aphilicity, BaseStaking, ZDNA and bDNATwist shows a cleft near TSS position. They indicated this region to be a putative promoter area. Abeel *et* *al* have also shown the relationship between structural profiles and known promoter elements, such as Tatabox, Initiator (which is mostly found at position +1 i.e., itself the TSS position), DPE and BRE [22]. They have demonstrated that the structural profile shows a specific peak and cleft in presence or absence of these primary sequence elements. For example, structural profiles are very similar for the promoters with or without the initiator (Inr) motif except a remarkable difference. For Inr containing promoters, a significant peak is visible on position −1, upstream of the TSS cleft, while this peak is missing in the promoter lacking the Inr motif. Based on these findings of the previous researchers, we have incorporated some rules that represent the features of a human core promoter region.

**Figure 2:**
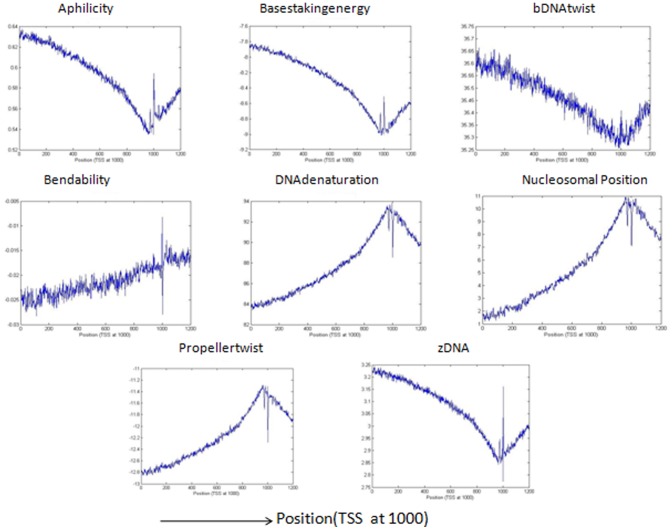
Structural pattern of eight different features for the sequences taken from UCSC table browser of hg19 human genome. The sequences used here are generated by grammars i.e. predicted as true promoter. The structural profiles are plotted with average value of window size 3 nt.

**Figure 3:**
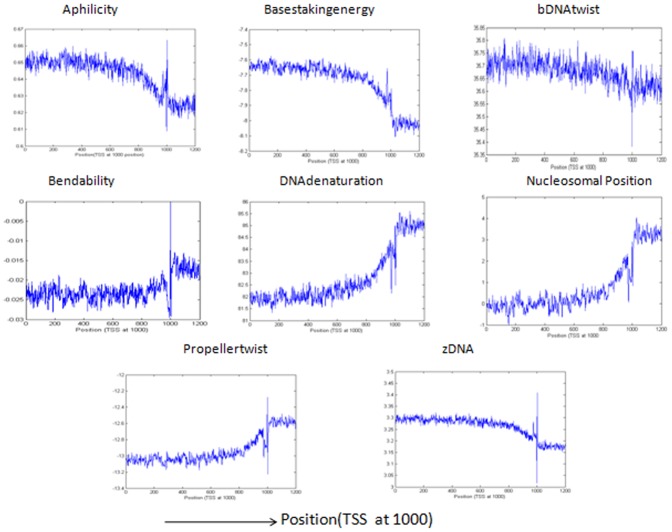
Structural pattern of eight different features for the sequences taken from UCSC table browser of hg19 human genome. The sequences used here are not generated by grammars i.e. redicted as nonpromoter. The structural profiles are plotted with average value of window size 3 nt.

**Figure 4:**
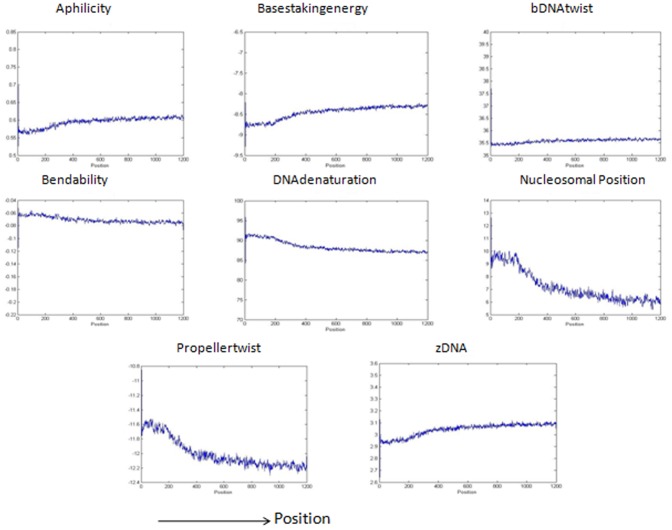
Structural pattern of eight different features for the nonpromoter sequences taken from EMBL and EID database. The sequences used here are predicted as true promoters by grammars. The structural profiles are plotted with average value of window size 3 nt.

**Figure 5:**
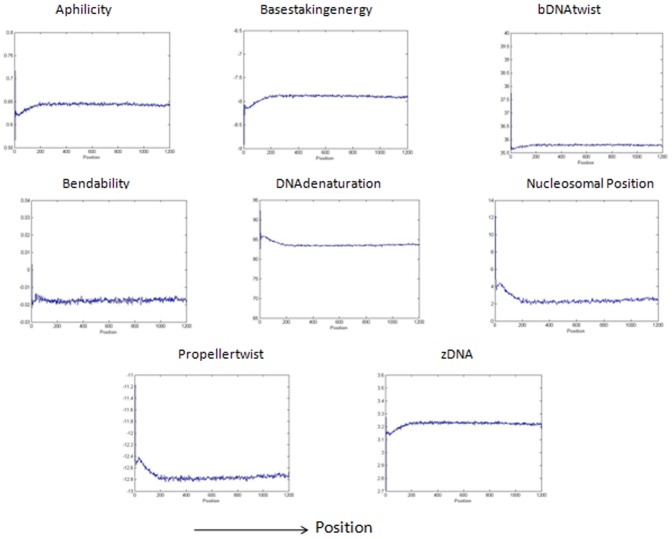
Structural pattern of eight different features for the sequences taken from UCSC table browser of hg19 human genome. The sequences used here are not generated by grammars. The structural profiles are plotted with average value of window size 3 nt.

If a sequence is annotated by the grammar rules as shown in **Table** **3**, then we have checked for all the structural profiles of that sequence. If the structural profiles show a high peak or cleft near the true TSS region, we have predicted the sequence as a promoter. We have also considered the structural profiles of that sequences which are not generated using the grammar rules; because, not all the promoters contain the signature elements in the sequences. This would minimize the false negative instances. In this case, if the structural profiles show the peak or cleft as per Abeel *et* *al* [22], we will predict it to be a promoter region. Sequences predicted as non-promoters if they are not generated by the grammar rules and also if the structural profiles of these sequences are not as that of a promoter region. In our method, the maximum allowed distance of the peak or cleft from the true TSS site is kept 500 bp for calculating the F-measure in contrast with other methods where the maximum allowed distance is 500 bp,1000 bp and 2000 bp [19], [20], [22]. **Fig.** **2** shows the structural properties of the DNA sequences taken from true promoter database that are generated by the grammar rules of **Table** **3**. The figure shows a high peak or cleft near TSS corresponding to the structural features. The structural features of the true promoter sequences that are not generated by the grammars are shown in **Fig.** **3**. The two figures clearly show the characteristic curve of structural features which represents a promoter sequence i.e., both the figures show a high peak and cleft near TSS region. **Fig.** **4** represents the structural features for the sequences taken from true non-promoter database, generated by grammar rules and **Fig.** **5** shows the structural features for the true non-promoter sequences not generated by the grammar rules. **Fig.** **4** and **Fig.** **5** explains the typical characteristic curve corresponding to the non-promoter sequences i.e., both the figures do not show any high peak or deep cleft in the curve. **Table** **4** illustrates the precision, recall and F-measure of sequences generated by the grammar rules for the two databases (dbtss, UCSC genome browser). **Table** **5** shows the prediction performance using grammar rules along with structural features. From these two tables, we may conclude that the performance of the system improves if we use both grammar rules and structural profiles to predict true promoters and non-promoters. All the structural features show a good balance between precision and recall. Grammar rules along with basestaking energy yields the best F-measure followed by Aphilicity. So grammar rule in conjunction with basestaking energy as structural feature is used for comparison of our method with existing promoter prediction tools.

**Table 4:** Prediction Performance of our proposed method using grammar rules on two datasets DBTSS and sequences from UCSC table browser for HG19.

Name of the Database	Precision	Recall	F-measure
DBTSS	0.66	0.57	0.61
Hg19	0.69	0.66	0.67

**Table 5:** Performance of grammar rule along with eight structural features of DNA on two datasets.

Property	UCSC genome browser(hg19)			dbtss		
	Recall	Prec.	F	Recall	Prec.	F
Basestaking energy	0.83	0.86	0.84	0.82	0.84	0.83
DNA denaturation	0.82	0.81	0.81	0.81	0.78	0.79
Nucleosome position	0.73	0.76	0.74	0.69	0.71	0.69
Aphilicity	0.87	0.80	0.83	0.84	0.79	0.81
bDNATwist	0.71	0.79	0.74	0.73	0.70	0.71
Propellertwist	0.80	0.74	0.76	0.77	0.73	0.75
zDNA	0.79	0.81	0.79	0.83	0.82	0.82
Bendability	0.69	0.70	0.69	0.74	0.75	0.74

Basestaking energy gives the best F-measure for all the dataset.

### Comparison with existing promoter prediction methods

To evaluate the prediction performance of our method, we have compared our method with four other open access human promoter prediction programs. We have selected these four methods among a number of promoter prediction tools because they have performed well in the previous comparative studies. The methods are FirstEF [52], Eponine [14], Ep3 [22] and ProSOM [53]. All these methods are free and publicly available. FirstEF is based on discriminant analysis to find potential first donor sites and CpG-related and non-CpG-related promoter regions. Ep3 uses large scale structural properties of DNA to locate promoter regions in whole genome sequence and ProSOM utilizes self-organizing maps to distinguish promoters from non-promoter sequences.

**Figure 6:**
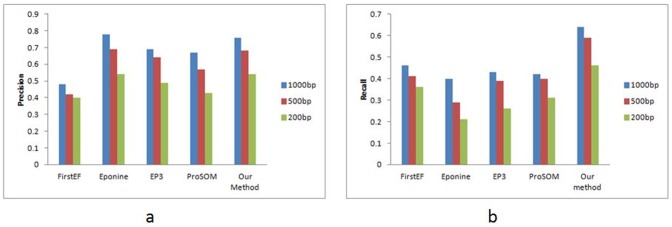
Genome wide precision and recall comparison with other methods. **a**) Comparison of genome wide precision value at different maximum allowed distances from annotated TSS (1000 bp, 500 bp and 200 bp). **b**) Comparison of genome wide Recall value at different maximum allowed distances from annotated TSS (1000 bp, 500 bp and 200 bp).

To avoid a biased evaluation of different promoter prediction methods, the performance comparison is conducted on the CAGE dataset. We apply our method and other methods to predict promoter on human genome. For our method, the DNA sequence of each chromosome is divided into a series of windows of length 1201 bp and step size 200 bp by sliding the window over the sequence. If a segment is predicted as promoter by our method, a possible TSS is marked. The noticeable TSS candidates falling within 1000 bp are merged into a cluster and the predicted output is the average of all the candidates in the cluster. Next the prediction result is evaluated with reference to the annotated TSS set of CAGE dataset. If a predicted TSS is within 500 bp of a true TSS position, then it is considered as a True TSS position i.e., true positive (TP). If a annotated TSS is not found in this region it is predicted as false negative (FN) and a prediction that lies inside the gene but not within the maximum allowable distance from true TSS, is false positive (FP). All the prediction methods used for comparison are tested with their default setting provided by their developers as the optimal parameters. The empirical results of comparisons are shown in **Table** **6**. To obtain unbiased result, we have also conducted the experiment with maximum allowable distance of 1000 bp and 200 bp. The precision and recall results of several promoter prediction methods at different maximum allowable distance from annotated TSS are shown in **Fig.** **6**. All promoter prediction methods except FirstEF give high precision values which indicate that the number of false positives is low. While Eponine has a considerably high precision but recall is very low leading to a low F-measure. On the contrarary, FirstEF achieves a balanced precision and recall but it shows a lower precision than others. EP3 and ProSOM also show a high precision and low recall but the balance of precision and recall is better than Eponine. From **Table** **6** and **Fig.** **6** we observe that the method proposed by us yields the best result with reasonably high and balanced precision and recall values resulting in yielding the best F-measure among all the methods. To sum up, our method outperforms all the methods used here for comparison for all the maximum allowed distances from annotated TSS and it can effectively distinguish promoter and non-promoter regions compared to other existing promoter prediction programs on a genome-wide scale.

**Table 6:** Genome-wide promoter prediction performance comparison of different promoterprediction programs with maximum allowable distance of 500 bp from annotated TSS.

Methods	Precision	Recall	F-measure
FirstEF	0.42	0.41	0.41
Eponine	0.69	0.29	0.40
EP3	0.64	0.39	0.48
ProSOM	0.57	0.40	0.47
Proposed Method	0.68	0.59	0.63

## Conclusion

In this communication, our analysis on promoter and non-promoter sequences gives an interesting finding from both structural and sequence perspective. The structural patterns are different for promoter and non-promoter sequences and the promoter region has a specific structural pattern. We have also shown that context free grammar rules can be generated to annotate a promoter region using pre-existing biological knowledge. Using these grammar rules, we are able to categorize the promoter and non-promoter sequences. Based on the above findings, we have developed a new method that utilizes context free grammar rules and structural features that can effectively predict promoter region. In contrarary to other methods, our method provides a good balance between precision and recall values and consequently improves the prediction accuracy for the whole genome level.

Our method is well-suited for human promoter prediction. Furthermore, our method can be applied for other species also. As a future work, we are trying to infer the grammar rules using different soft computing approaches for the promoter regions of other species and also for different genomic sites and protein sequences for which we don't have any pre existing knowledge relative to the structure of the sequence elements.
